# Health professional’s perception of a smoking cessation intervention among disadvantaged patients participating in a pragmatic randomized trial

**DOI:** 10.1186/s12913-023-09950-2

**Published:** 2023-09-14

**Authors:** Aurélia Manns, Sarah Mahdjoub, Gladys Ibanez, Emilie Jarrier, Ava Daeipour, Maria Melchior, Fabienne El-Khoury

**Affiliations:** 1 Department of social epidemiology, Sorbonne Université, Institut Pierre Louis d’Epidémiologie et de Santé Publique, INSERM, IPLESP, 27 rue Chaligny, Paris, 75012 France; 2 Faculty of Medicine Pierre et Marie Curie, Department of Education and Research in General Medicine, Sorbonne Université, Paris, F75012 France

**Keywords:** Smoking cessation, Low socioeconomic position, Perceived barriers, Research intervention implementation, Health professionals, Pragmatic trials

## Abstract

**Introduction:**

Individuals who have a low socio-economic position (SEP) are more likely to smoke and face greater barriers to quitting tobacco. However, the effectiveness of tailored interventions has been limited probably due to specific challenges relative to this population. We conducted a mixed-method study to better understand health professionals’ perceptions and barriers when implementing a preference-based smoking cessation (SC) intervention among disadvantaged smokers.

**Methods:**

A self-administered online questionnaire was sent to health professionals (doctors’ and other health professionals specialized in SC) participating in “STOP” a pragmatic multicentre randomized controlled trial. Perceptions regarding patient eligibility, the doctor-patient relationship, general study organization, and satisfaction were measured.

**Results:**

Twenty-eight STOP study investigators responded. Health professionals prioritize smoking cessation for disadvantaged patients, but face challenges in approaching and following them. A research intervention providing cessation tools based on preference was deemed useful but generally undermined by time constraints. Health professionals’ preconceptions regarding patients in low SEP having other “pressing problems” which might be exacerbated by quitting smoking were also identified. Further, participation in a research intervention was perceived as not satisfactory due to workload and lack of time.

**Conclusion:**

Our results highlight general barriers inherent to implementing pragmatic trials. They also present specific challenges in smoking cessation trials among disadvantaged population, essential to advance equity in tobacco control.

**Supplementary Information:**

The online version contains supplementary material available at 10.1186/s12913-023-09950-2.

## Introduction

Smoking is a major public health concern and one of the leading causes of preventable death and disease worldwide [1]. Despite multiple tobacco-control efforts, smoking prevalence remains high in most Western countries, particularly among individuals in low socio-economic positions (SEP) [2]. In France, almost one adult in 4 (25.3%) smokes cigarettes daily, with a higher prevalence in people in low SEP (32% of French adults with no high school diploma smoke) [3]. Individuals with socioeconomic disadvantages are not only more likely to smoke, they also have higher rates of nicotine dependence, and face greater barriers to quitting smoking than those who belong to higher socio-economic backgrounds [4].

Hence, tailored interventions were developed to support smoking cessation among disadvantaged populations [5]. However, these interventions face specific challenges when working with individuals with low SEP, who often have several concomitant medical and psychosocial needs that can make it difficult for health professionals to support them in quitting smoking [6, 7].

Smokers with socioeconomic disadvantages report lower quitting rates compared to more affluent smokers, regardless of the smoking cessation intervention method [8, 9]. Combining pharmacotherapy and structured behavioral support has shown some success in helping smokers with socioeconomic disadvantages quit, but their quit rates are still lower than those of more affluent smokers. Lack of support for quit attempts, stronger nicotine dependence, lower motivation to quit, and less compliance with treatment are other possible reasons explaining why quitting is more difficult for low SEP smokers. Disadvantaged smokers may also have less support from their family or community because they are more likely to have smokers in their social network. Motivation, use of pharmacotherapy and smoking cessation services, and the nature of smoking cessation programs can also affect quitting success, but inconsistent findings have been reported [8]. A systematic review by Kock et al. (2019) [10] indicates that individual-level interventions can be effective in helping disadvantaged smokers quit. However, tailoring these interventions specifically for disadvantaged smokers did not show significant benefits [10]. The authors concluded that to achieve positive smoking cessation outcomes for this group, it may be necessary to improve the development of more equitable interventions that are better tailored to their needs.

There is evidence that smoking cessation interventions in primary care settings can be effective in reducing tobacco-related health inequalities [11], however, these types of interventions are under-utilised [12]. Moreover, delivering smoking cessation interventions in low SEP populations presents specific challenges, particularly in the context of a research trial. These challenges include issues such as low literacy, lack of trust in the medical system, and difficulties in communication [13]. Identifying such barriers can inform the development of more effective smoking cessation interventions for disadvantaged populations, as well as provide guidance for researchers and tobacco-control health professionals.

To address these challenges, our team launched a pragmatic randomized controlled trial (RCT) assessing the effectiveness of a preference-based smoking cessation intervention centred on the patient’s preference : the STOP (Sevrage Tabagique à l’aide d’Outils dédiés selon la Préférence: Smoking cessation using preference-based tools) RCT. Participants randomized to the intervention group of this RCT receive smoking cessation tools delivered free of charge by a health professional, according to their choice. Doctors and other health professionals specialized in smoking cessation are responsible for enrolling and randomizing eligible patients, as well delivering the intervention in both arms [14]. As a pragmatic trial, the intervention was designed to be integrated into routine care and approximate a real-life setting.

The objective of this pragmatic trial is to determine treatments’ effects in ‘real-life’ conditions, by getting as close as possible to the actual conditions under which the treatment could be implemented. Health professionals who usually participate in pragmatic trials are chosen not because they are specialists in their field but because they conform to usual care. Thus, understanding the perceptions and barriers to implementation of this intervention among health professionals is essential to optimize both research and routine practices [15]. The present analysis aims to better understand the challenges and barriers faced by health professionals when implementing smoking cessation interventions among low SEP individuals in a research context.

This article therefore presents findings based on a questionnaire administered to these health professionnals, and provides a critical reflection on the challenges of implementing smoking cessation interventions among low SEP individuals in ‘real-life settings’.

## Methods

### STOP : study design and outcomes

The design of the STOP pragmatic multicenter randomized controlled trial (RCT) is described in more details elsewhere [16]. Trial registration number NCT04654585.

The primary outcome is smoking abstinence at 6 months after inclusion, defined as self-reported continuous abstinence for at least 7 days. Secondary outcomes include the total number of days of abstinence at 6 months after inclusion, continuous abstinence for at least 7 days at one and three months after inclusion and number of relapses.

#### Participants in the STOP RCT

The main inclusion criteria is regular smoking of at least five cigarettes per day, a willingness to quit or reduce tobacco consumption, and a low socio-economic position (low SEP). To assess SEP, individuals had to be either unemployed or eligible for a social benefit reserved to low-income individuals living in France. Participants also have to be at least 18 years of age, available for four follow-up appointments over the course of six months, and covered by the French national health insurance system. Patients who are already attempting to quit smoking, or those under guardianship or legal curatorship were not eligible to be included, as were persons who did not speak French.

#### Study centers and recruitment – STOP RCT

The study is being implemented in eighteen medical centers in France, including eight in the greater Paris area, four in Lyon and six in other cities as presented in supplementary Figure S1. Participating medical centers include public hospitals, municipal health centers, or addiction treatment and prevention facilities, which concentrate a high proportion of patients with low SEP. The recruitments are carried out by physicians (general practitioners, addiction specialists, or smoking cessation specialists), or other health professionals specialized in smoking cessation (i.e. nurses or pharmacists). After receiving oral information about the study protocol, individuals who agree to participate are asked to answer a pre-inclusion questionnaire, and, if eligible, to sign a written informed consent. They are then randomized into two groups (control group vs. intervention group). The study is conducted in a single-blind fashion.

#### Ethical approval

The study protocol was approved by a French Ethical Committee (CPP) - n°: 20.01.31.65528 RIPH2 HPS.

#### STOP intervention

Participants in both groups are treated for smoking cessation according to the latest recommendations and usual medical practice [17]. In addition, patients in the intervention group directly receive free Nicotine Replacement Treatment (NRT: patches, inhalers, gum, sublingual tablets, and lozenges) and/or an electronic cigarette (supplied with e-liquid), according to their preference. A brief and clear description of each smoking cessation tool is given by the health professional and, depending on the participant’s subsequent choice, a sufficient quantity of the tool or tools of choice is provided to last until the next appointment.

### Health professionals’ questionnaire

An email containing a link to an online questionnaire on the Lime Survey platform was sent to each healthcare professional participating in the STOP RCT, months after recruitment started in their center. The questionnaire examined perceptions regarding four possible barriers or facilitating factors in the trial’s implementation:

Patient eligibility: Health professionals were asked how often they met ‘eligible’ patients (i.e. persons who smoke, are willing to stop smoking, and have a low SEP). Practitioners’ routine practices were also examined, by asking if they generally brought up smoking and if they thought that smoking cessation for low SEP patients was a priority.

Doctor-patient relationship: Health professionals were asked whether, compared to other patients, low SEP patients were generally different in terms of patient-physician trust, adherence to care, regular follow-up, difficulty in undertaking long consultations, adherence to treatment, follow-up, addressing the issue of smoking cessation, and participating in a research study.

Study organization: participants’ views on difficulties (ranging from very simple to very complex) regarding the evaluation of eligibility criteria and the RCT’s integration into clinical practice were assessed. Difficulties related to time constraints were also assessed, by asking how much time the study added per week in terms of: 1/ paperwork, 2/ study presentation to patients, and 3/additional appointment time (ranging from less than ten minutes to more than two hours).

Satisfaction: we also examined health practitioner’s perceptions of the research’s merits and whether participating in the study had benefits for them in terms of: 1/ more frequent discussions relative to smoking cessation, 2/ new knowledge about NRT and e-cigarettes, and 3/ changes in perceptions of smoking cessation among patients with socioeconomic disadvantages. They were also asked if giving out free smoking cessation tools made them feel like they were providing a better treatment than a prescription, or resulted in better patient compliance. Finally, a question was asked about possible treatment contamination among patients part of the intervention and control groups (the study being single blind).

Most of the questions followed the Likert scale [18].

We also collected data regarding health professionals’ sex, age, occupation (nurse specialized in smoking cessation, general practitioner, doctor specialized in smoking cessation, addictologist doctor, other) and smoking status. Occupations were classified into two categories: doctors and other health professionals (according to their profession). We also identified smoking cessation specialists (‘tobbacologist’ Yes/No).

#### Open-ended questions

**Fig. 1:**
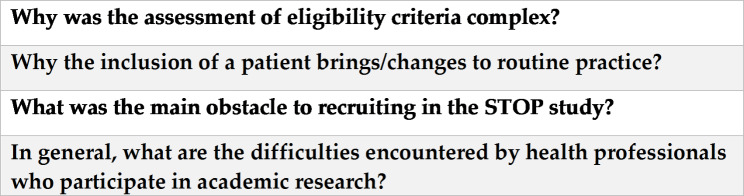
Open ended questions addressed to health professionals participating in the STOP RCT

Four questions in the online questionnaire were open-ended with no character limits. They are presented in Fig. 1.

### Analyses

All statistical analyses performed were descriptive. The frequencies of each response were calculated and compared across different categories of health professionals (occupation, specialty, health professional’s smoking status) when relevant. All analyses were conducted using SAS 9.2.

## Results

Twenty-eight health professionals participating in the STOP study completed the questionnaire. The mean age was 47 years old (sd = 10). Their characteristics are presented in Table 1.

**Table 1:** Characteristics of health professionals participating in the STOP RCT. (France 2023)

Characteristics	Number of participants N = 28 N(%)
Sex	
Men	7 (25)
Women	21 (75)
Occupation	
Medical Doctor	17 (60.7)
Other health professionals specialized in smoking cessation	11 (39.3)
Smoking cessation specialist	
Yes	17 (60.7)
No	11 (39.3)
Smoking status	
Smoker	2 (7.1)
Former smoker	10 (35.7)
Non smoker	16 (57.1)

### Health professional’s perceptions

#### Patient’s eligibility

As shown in supplementary Table s1, health professionals declared that they are often treating patients with a low SEP. They also reported that the majority of their patients are smokers who are not spontaneously requesting to quit smoking and who are not yet treated for smoking cessation.

**Fig. 2:**
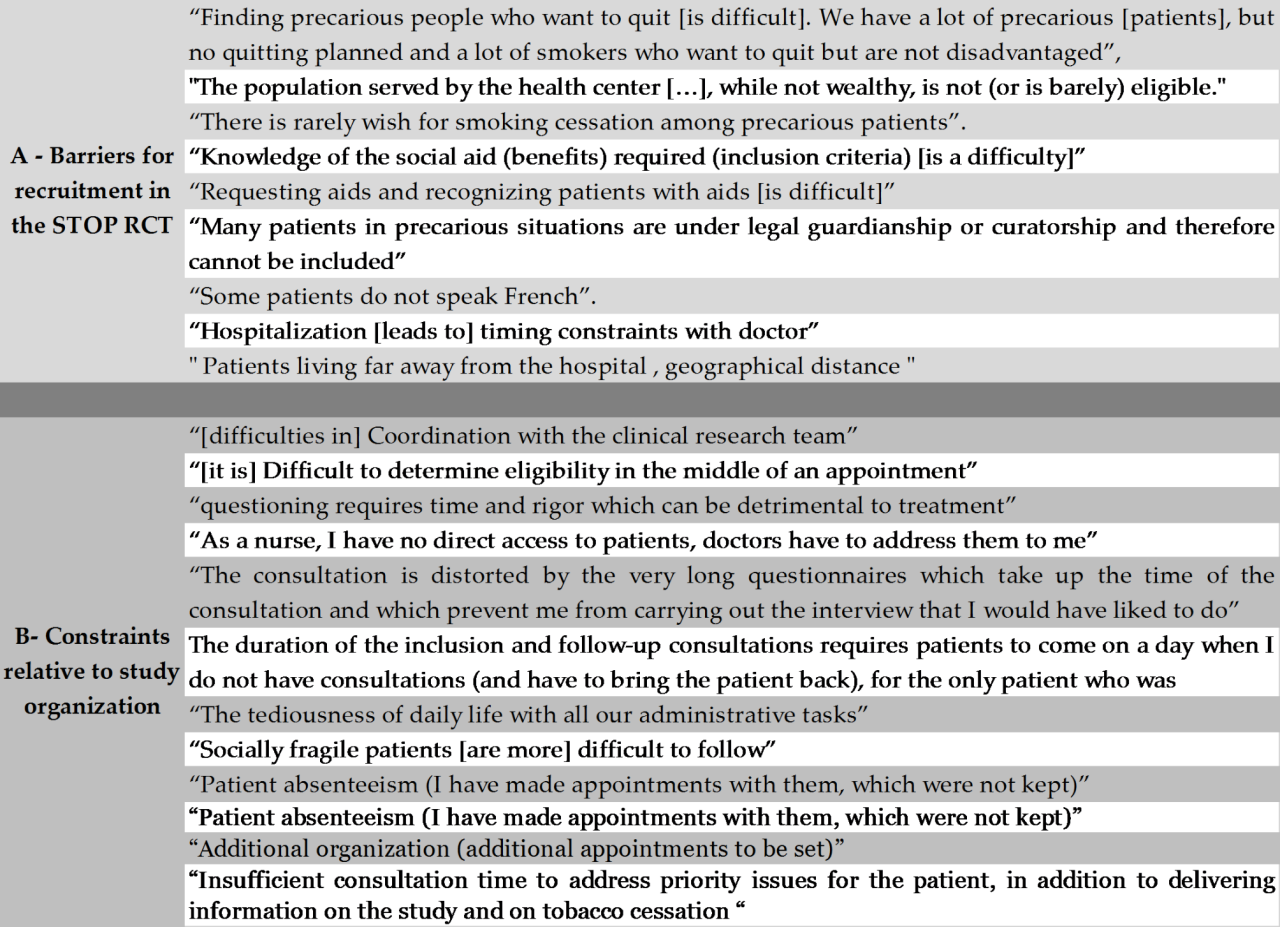
Perceptions reported health professionals participating in the STOP RCT concerning barriers for recruitmenet, and constraints relative to study organization

Participating health professionals indicated that many of their patients met eligibility criteria for the STOP RCT. However, several comments on barriers for recrutment were reported in Fig. 2-A.

For tobacco-cessation specialists other types of barriers to recruitment were identified:

“We are already doing too much smoking cessation (we are even proactive; we teach medical students on rotation to prescribe patches to smokers who come for bronchitis, even if they don’t ask for it, in the belief that this proactive approach described in some studies will lead patients to at least try and discuss the experience with a doctor). Therefore, recruitment [for the STOP RCT] is not easy. In the health center where we already do a lot of smoking cessation (the doctors and the specialized nurse); thus the majority of eligible patients encountered already have patches or had already been offered them in the past year.”

“Very often my patients are already on NRT so it is rather difficult to recruit new ones.”

#### Doctor-patient relationship

**Fig. 3:**
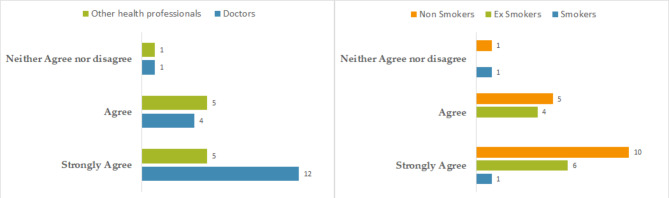
Health professionals participating in the STOP pragmatic RCT perceptions concerning “Priority given to smoking cessation in low SEP patients” according to the health professionals’ profession and smoking status (France, 2023)

Health professionals mostly agreed with the statement “As a professional, I think that smoking cessation is a priority for my patients with low SEP”. Some differences can be observed depending on the health professional’s occupation, and depending on their smoking status, as described in Fig. 3.

Compared to other patients, initiating a smoking cessation for a disadvantaged patient was perceived as more difficult. Participating in a research study was also perceived as less convenient when participants are in low SEP. Interviewed health professionals also reported that it was more difficult to schedule multiple appointments (i.e. asking the patient to come back only due to the study), follow-up was less consistent, and treatment adherence was perceived as lower than among patients with high SEP (Table 2).

**Table 2:** Perceived differences between low and high SEP patients as ascertained by health professionals participating in the STOP RCT (France, 2023)

	Superior N (%)	Comparable N (%)	Inferior N (%)
Ease in making multiple appointments	3 (10.7)	8 (28.6)	17 (60.7)
Difficulty to carry out long appointments	4 (14.3)	15 (53.6)	9 (32.1)
Ease in regular follow-up	0	4 (14.3)	24 (85.7)
Treatment adherence	2 (7.1)	11 (39.3)	15 (53.6)
Patient-Physician trust	7 (25)	15 (53.6)	6 (21.4)
Convenience to initiate smoking cessation	1 (3.6)	14 (50)	13 (46.4)
Convenience to initiate study participation	1 (3.6)	12 (42.9)	15 (53.6)

#### Satisfaction

Participating health professionals were mostly convinced of the merits of the STOP RCT. However, they did not report being more interested in e-cigarette or NRT because of the study, and not having discussed smoking cessation more frequently with their patients. Their perception of the low SEP patients’ desire to stop smoking has not changed compared to their views prior to study participation (supplementary table s2).

**Fig. 4:**
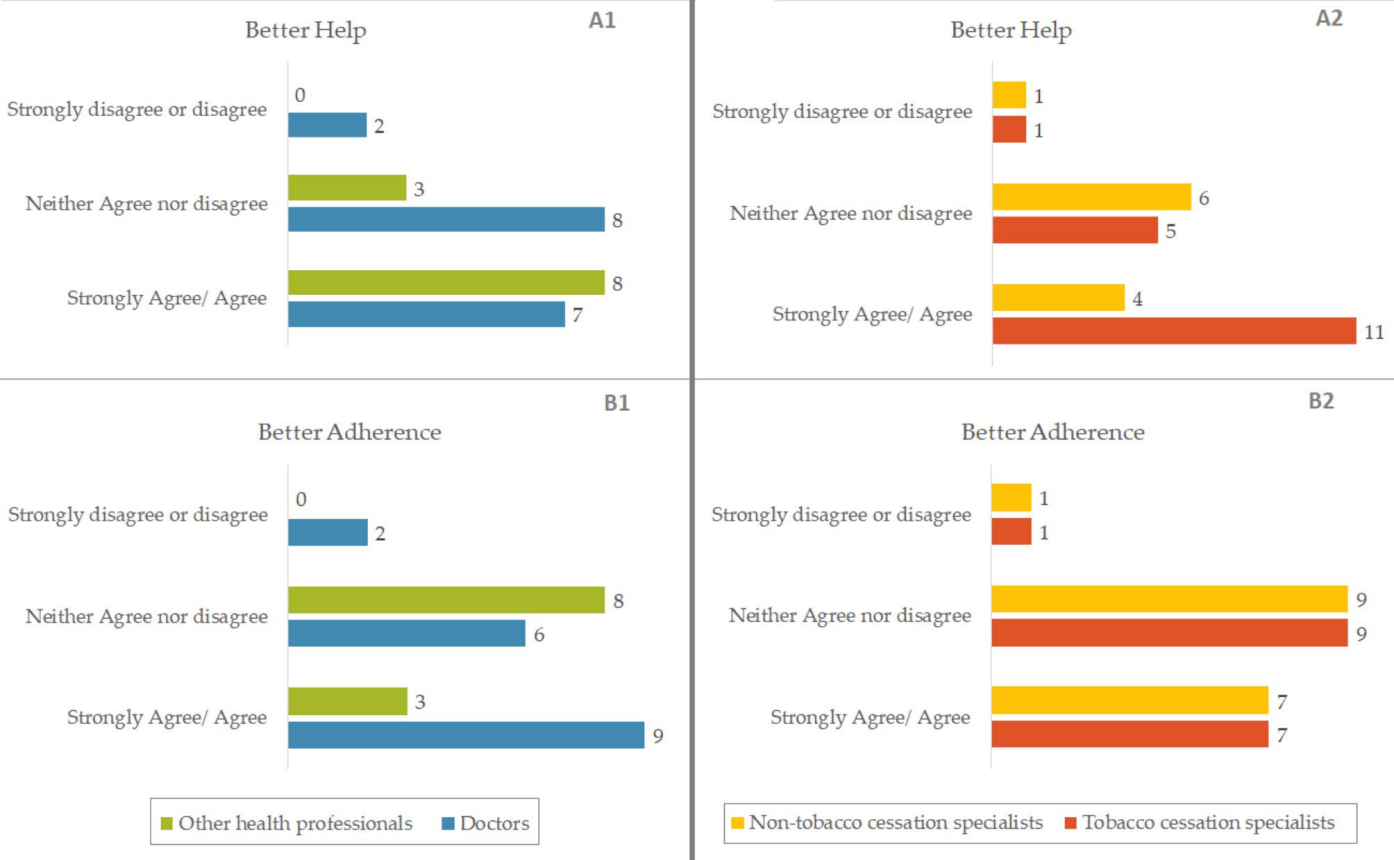
Answers to the questions « Giving products free of charge allowed me to provide a better help than with a simple prescription (**A**) » and « Giving products free of charge allowed patients to have a better adherence to treatment (**B**) », according to profession or specialty of health professionals participating in the STOP RCT.(France, 2023)

Figure 4 illustrates perceptions regarding the delivery of free smoking cessation tools according to the occupation or specialty of the health professional. Compared to other health professionals specialized in smoking cessation and non-smoking cessation specialists, doctors and smoking cessation specialists adhere more to the fact that giving free products allows better treatment adherence. Similarly, they report feeling that they are better able to help patients by giving free nicotine replacement products than by simply prescribing them.

#### Study organization and time constraint

Overall, the study took approximately 10 min to present the aims 30 min to explain the procedures in addition to a standard appointment time, 30 min of time dedicated to administrative procedures and data colelction (Table 3). Time is the most frequent constraint to study participation reported by participating health professionals. This was in fact mentioned in almost half of the open-ended comments (31 out of 81).

**Table 3:** Additional time per week due to participating in the STOP RCT reported by health professionals implementing the trial

	Consultation time N (%)	Presentation of the study N (%)	Administrative procedures N (%)
< 10 min	5 (17.9)	16 (57.1)	8 (28.6)
10–30 min	9 (32.1)	11 (39.3)	11 (39.3)
30 min-1 h	11 (39.3)	1 (3.6)	6 (21.4)
1-2 h	3 (10.7)	0	3 (10.7)
> 2 h	0	0	0

In addition, the other reported constraints relative to study organization are presented in Fig. 2-B.

But it also has been reported that participation in the study can have positive impacts on routine care and practices:

“[The intervention] helps address aspects of perception of nicotine substitutes and e-cigarettes that are not necessarily spontaneously addressed. It also makes it easier to discuss questions of preference related to the taste of NRTs, the concentration of e-liquids.“,

“An improvement in my patient-centered approach”.

Regarding participation in an academic research study, the following remarks were noted by participating health professionals:

“The times of care and research should be seperated”.

“Multiplicity of studies with the fear of making mistakes and distorting the study”.

“The overall workload at the public hospital, any study requires additional explanations and lengthens consultations”.

“Several patients did not wish to participate in the study on principle, as they did not want to be included in a research protocol, even after I explained that it would not change anything in their treatment. Moreover, it is sometimes difficult to explain to patients the benefits of the study (their interest as patients) without mentioning the two randomized groups (without telling them that they may potentially receive free treatments).“

“The act of participating in an interventional study modifies practices (in any arm, for that matter).“

“The training for field research in primary care is not widely disseminated, and still not sufficiently integrated into practice. However, the daily operational question remains: on-site support from clinical research assistants would help ensure the proper conduct of research and a form of acculturation for primary care physicians.“

The contribution of a clinical research assistant was noted three times as being valuable.

## Discussion

Health professionals in our study sample routinely meet low SEP patients who smoke, and consider it a priority to offer them smoking cessation treatment. However, they are confronted with challenges specific to these patients, with whom the uptake of smoking cessation, regular follow-up, and adherence to care are perceived as complex.

We also highlighted perceived obstacles to study implementation that are inherent in the study organization and design, mainly due to time constraints. However, health professionals were mostly convinced of the usefulness of this research and felt they were providing better help through the intervention, by delivering smoking cessation tools free of charge according to the patient’s choice, than with a standard prescription. In clinical practice outside of a research trial, this intervention does not present the same type of administrative time constraints, but it does require consultation time dedicated to explaining the smoking cessation tools that the patient has at his disposal so that he or she can make an informed choice.

### Interpretation

#### Barriers to patients recruitment

Patients received in consultation by health professionals mostly correspond to the target population of our study, however several difficulties relative to recruitment were reported, which can be grouped into four categories:

Exclusion criteria (lack of French language skills, a high income, a center policy that results in patients being mostly already treated, under legal guardianship),

Difficulties to evaluate eligibility criteria (detecting social benefits/ low SEP),

Problems inherent to the patient (hospitalized, geographical distance),

And finally, some health professionals’ preconceived idea that the desire to stop smoking is not a priority for smokers with low SEP, and quitting is perceived as more challenging among this population.

This last point calls for vigilance regarding the prejudices that some health professionals might have relative to what the patient thinks is a priority for him/her.

Although health professionals agreed that smoking cessation is a priority for their low-SEP patients, some comments indicated otherwise.

A study based on the National Health Interview Survey (NHIS) of 2001 in the US [19], revealed that assistance with smoking cessation was less frequently reported by patients with high or mid-levels of disadvantage compared to those with low levels. Levels of disadvantage were scored depending on education, income, and health insurance. The authors explain this by the fact that patients in these situations are those among whom prevalences of smoking and associated comorbidities are the highest. Thus, health care professionals may feel that they are less likely to be successful in quitting. In addition, the facilities that provide care for this patient population are often in busy, under-staffed environments, which limit the smoking cessation treatments.

A systematic review on perceived barriers to smoking cessation among vulnerable groups reports cases where health professionals discourage smoking cessation because they were concerned about their mental health, or because they thought smoking was their only source of pleasure [20]. Several studies cited in this review highlight lack of support to quit from health professionals [20]. These results based on a systematic review of the literature suggest that some health professionals might have the preconception that disadvantaged patients have other “higher priority” problems and that quitting smoking could add to or exacerbate them.

Finally, health professionals report that smokers with low SEP have inherent difficulties in smoking cessation. Withdrawal is perceived as more complicated, as is smoking cessation initiation or participation in a study. Follow-up and adherence to care are also perceived as more difficult for these patients.

A literature review on barriers faced by low SEP patients to smoking cessation reports a lower ability to reach smoking cessation support because of low mobility, low social support, and a tendency to perceive it as inappropriate or inaccessible [21].

#### Participation of health professionals in a research intervention

Health professionals did not report high satisfaction with their participation in the study. They mostly did not report gaining more information about NRT and e-cigarettes, discussing smoking cessation more often, or changing their perception concerning the desire to quit among low SEP populations. However, one of the goals of the study is to prove that giving free cessation tools to these patients according to their own choice would lead to greater success in quitting smoking. The professionals most convinced that this intervention would allow them to better help their patients, or would allow a better adherence to the care of their patients, were the smoking cessation specialists and the medical doctors.

The most frequently cited barriers and constraints related to participating in the study were the lack of time combined with the already present heavy workload. Indeed, since the investigating centers are public hospitals or health centers, the lack of staff and the influx of patients beyond capacity can make research take second place to care. Consultations must be quick and efficient in order to see as many patients as possible, and the addition of time that is not strictly necessary is perceived as burdening and therefore denied. The SESMAT qualitative survey [22] asked physicians about work difficulty. The results highlight physicians’ frustration over chronic extensive workload (quantity, time, lack of recognition), administrative burden and lack of time. Since these issues are already present in everyday life, the addition of a research intervention can represent a constraint rather than a motivation.

Health professionals may also have been uncomfortable by the content of some questions in the questionnaire, such as questions on socio-economic background by fear of stigma. They reported that it could be complicated to submit it into the middle of a specific appointment, and that it may strain the already fragile therapeutic alliance with low SEP patients. Moreover, due to lack of time, sometimes these questionnaires replaced routine questioning that the professional would have liked to carry out. Thus, it may be considered more relevant to separate the time of care and the time of research by avoiding integrating the data collection in the consultation. In addition, they may be apprehensive about patients’ reluctance to participate in research. Patients may experience it as a “test”, and this may weaken the physician-patient trust relationship. Finally, health care professionals are sometimes not well trained in research and may be afraid of making errors and distorting the study.

Our results are consistent with a qualitative study on perceptions of recruiters in six Randomized Control Trials (RCTs) [23]. Doctors struggled to balance their roles as recruiters and clinicians, leading to conflicts with clinical practice, uncertainty about the best treatment, and willingness to recruit patients. Nurses also faced conflicts between their roles as caring clinical nurses, patient advocates, and recruiters/scientists, causing considerable discomfort and difficulty. Organizational difficulties, lack of eligible patients, and patients’ strong treatment preferences were identified as recruitment barriers, but they were not straightforward and were often reinforced by recruiters’ views. Recruiters’ discomfort and difficulty were significant, leading to low morale and poor recruitment levels, as they had conflicts between carrying out research and ensuring individual patients’ best interests, and strong treatment preferences or clinical instincts that made them uncomfortable recruiting patients outside their comfort zone.

Our results highlight the desire of some health professionals to separate research time from clinical practice time. However, the aim of this pragmatic research, and therefore of the pragmatic trial, was to integrate research into routine, so that the intervention would be as close as possible to real-life conditions. This is so the intervention could be more easily generalized outside the research context.

Some professionals did have positive feedback on the integration of the intervention into their medical practice: it allowed them to refocus on patient preferences and thus improve their patient-centered approach to care. The co-construction of interventions with the target audience is indeed a criterion of effectiveness according to a synthesis of the literature conducted by Guignard et al. on smoking cessation interventions in low SEP patients [24]. The value of relying on patient preference has already been explained in the STOP study protocol justification [16].

A qualitative study asked key stakeholders about the challenges raised by pragmatic trials [25]. It was noted that the difference between research, quality improvement, and practice is sometimes unclear. The boundary is also not well defined for the patient during the consultation time (the point at which one moves from care to research). This may explain why the health professionals in our study would prefer to separate the two components. Moreover, it is requested to compare the intervention to usual care. which can differ between each patient, and between each caregiver. Therefore the difference between the intervention and usual care may vary within the same study. The workload generated by the administrative aspect of the research would then seem disproportionate, for a lesser addition to the usual care given.

Finally, in real life, health professionals are confronted with specific problems while treating low SEP patients. This is why a pragmatic trial specifically tailored to this population has been set up. However, these same issues recur as barriers to the implementation of the research, which illustrates all the complexity of pragmatic trials not just those targeting individuals with lown SEP.

### Limitations

Despite the valuable insights provided by this study, there are some limitations that should be acknowledged. Firstly, the sample size was relatively small, with only 28 doctors and other health professionals specialized in smoking cessation participating in the study. This limited sample size may affect the generalizability of the findings and the representativeness of the sample.

Secondly, the study was conducted in France, which has a unique healthcare system and cultural context. This may limit the applicability of the findings to other countries with no universal health care system.

Thirdly, the questionnaire used in this study was auto-filled, which may have led to some bias in the responses. While the use of an auto-filled questionnaire ensured consistency in the responses, it may have limited the depth and richness of the data collected.

Finally, the recruiters who participated in the study were not working in the same conditions. This may have influenced their perceptions and experiences with delivering the intervention and may have affected the results.

Addressing these limitations in future studies will enhance our understanding of the issues facing disadvantaged populations and inform the development of more effective smoking cessation interventions.

## Conclusions

This study highlights the challenges and barriers faced by health professionals when implementing smoking cessation interventions among low SEP individuals in a pragmatic trial context. A patient-preference intervention where smoking cessation tools were freely delivered was seen as useful and effective, but several challenges limited recruitment and implementation.

The findings suggest that while health professionals recognize the importance of offering smoking cessation interventions to this population, they face specific constraints in their routine that must be addressed. There is a need to address preconceptions and a better understanding of the health problems of low SEP patients. An improvement of research training among health professionals would help integrate the time of research in their daily practice in order to ensure successful implementation of such interventions, without having to separate time of research and time of medical consultation.

The insights gained from this study can inform the development of more effective smoking cessation interventions for disadvantaged populations, as well as provide guidance for researchers and health professionals working in this field, notably those looking to set up pragmatic trials which are increasingly encouraged.

### Electronic supplementary material

Below is the link to the electronic supplementary material.

Supplementary Material 1

## Data Availability

Available upon request from corresponding author, at fabienne.khoury(at)inserm(.)fr.

## List of Abbreviations

CPP: Comité de Protection de Personnes
INCA: Institut National de Cancer
NHIS: National Health Interview Survey
NRT: Nicotine Replacement Treatment
RCT: Randomized controlled trial
SEP: Low socio-economic position
STOP: Sevrage Tabagique à l’aide d’Outils dédiés selon la Préférence:Smoking cessation using preference-based tools
Sd: Standard deviation
Ref: Reference category
