# Supplementary material for: Health professional’s perception of a smoking cessation intervention among disadvantaged patients participating in a pragmatic randomized trial
Source: BMC Health Serv Res. 2023 Sep 14;23:993. doi: 10.1186/s12913-023-09950-2 (PMC10503146; doi:10.1186/s12913-023-09950-2)
Supplement: Supplementary file 1 — Supplementary Material 1 [file 12913_2023_9950_MOESM1_ESM.docx]

Supplementary files


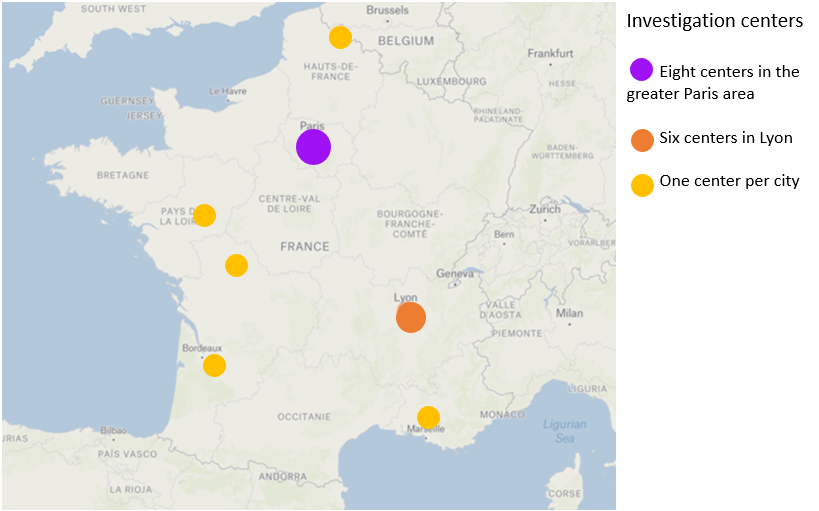


**Figure S1: Map of the investigation centers participating in the STOP RCT. France**

|  | **Smokers N(%)** | **Smokers requesting to quit N(%)** | **Smokers treated for smoking cessation N(%)** |
| --- | --- | --- | --- |
| Majority | 12 (44.4) | 3 (11.11) | 2 (7.4) |
| More than half | 2 (7.4) | 1 (3.7) | 2 (7.4) |
| Half | 5 (18.5) | 7 (25.9) | 4 (14.8) |
| Less than half | 8 (29.6) | 9 (33.3) | 7 (25.9) |
| Rarely | 0 | 7 (25.9) | 12 (44.4) |
|  | Low income patients N(%) | | |
| Very often | 7 (25.0) |  |  |
| Often | 16 (57.1) |  |  |
| Sometimes | 5 (17.9) |  |  |
| Rarely or Never | 0 |  |  |
|  |  |  |  |

**Table s1 : Patients’ characteristics according to health professionals perceptions**

|  | **Strongly agree or Agree**  **N (%)** | **Not agree or disagree**  **N (%)** | **Strongly disagree or disagree N (%)** |
| --- | --- | --- | --- |
| **Allows to develop interest in e-cig or NRT** (n=28) | 7 (25) | 5 (17.9) | 16 (57.1) |
| Smoking cessation specialists (n=17) | 6 (35.3) | 1 (5.9) | 10 (58.8) |
| Non- smoking cessation specialists | 1 (9.1) | 4 (36.4) | 6 (54.6) |
| **Allows to approach SC more often** (n=28) | 6 (21.4) | 7 (25) | 15 (53.6) |
| Smoking cessation specialists (n=17) | 4 (23.5) | 2 (11.8) | 11 (64.7) |
| Non-smoking cessation specialists (n=11) | 2(18.2) | 5 (45.5) | 4 (36.4) |
| **Convinced in merit and utility of research** (n=28) | 25(89.3) | 3 (10.7) | 0 |
| **Changed perception on smoking cessation wish** (n=28) | 5(17.9) | 8 (28.6) | 15 (53.6) |

**Table s2 : Health professionals’ satisfaction concerning the STOP RCT (France, 2023).**
